# Arterioportal Fistulas (APF) in Liver Tumors: Prognosis in Relation to Treatment

**DOI:** 10.1155/1992/47601

**Published:** 1992

**Authors:** Anatoly M. Granov, Pavel G. Tarazov, Vladimir K. Ryzhkov

**Affiliations:** Central Research Institute of Roentgenology and Radiology ul. Leningradskaja 70/4 Pesochny-2 Leningrad 189646 Russia

## Abstract

Prognosis of 16 patients with hepatic tumors and angiographically proven arterioportal fistulas was analysed in relation to treatment. Six patients received only conservative therapy; they all died of variceal bleeding in the course of two months after angiography. Hepatic resection was performed in four patients; three of them are still alive 13–52 months later including two free of both the tumor and portal hypertension. Hepatic artery embolization was carried out in six patients. All of them died in 2–36 months after the procedure, but only two from gastroesophageal hemorrhage. It is concluded that prognosis of arterioportal fistulae in liver neoplasms is poor due to hyperkinetic portal hypertension and following variceal bleeding. Hepatic resection of both the tumor and the fistula is the treatment of choice. In unresectable cases hepatic artery embolization will decrease the risk of variceal hemorrhage.


*HPB Surgery*, 1992, Vol. 5, pp. 87–94
Reprints available directly from the publisher
Photocopying permitted by license only

© Harwood Academic Publishers GmbH
Printed in the United Kingdom

### ARTERIOPORTAL FISTULAS (APF) IN LIVER TUMORS: PROGNOSIS IN RELATION TO TREATMENT

ANATOLY M. GRANOV, PAVEL G. TARAZOV and VLADIMIR K. RYZHKOV

*Central Research Institute of Roentgenology and Radiology, Leningrad, USSR*

*(Received 3rd April 1991).*

Prognosis of 16 patients with hepatic tumors and angiographically proven arterioportal fistulas was analysed in relation to treatment. Six patients received only conservative therapy; they all died of variceal bleeding in the course of two months after angiography. Hepatic resection was performed in four patients; three of them are still alive 13–52 months later including two free of both the tumor and portal hypertension. Hepatic artery embolization was carried out in six patients. All of them died in 2–36 months after the procedure, but only two from gastroesophageal hemorrhage.

It is concluded that prognosis of arterioportal fistulae in liver neoplasms is poor due to hyperkinetic portal hypertension and following variceal bleeding. Hepatic resection of both the tumor and the fistula is the treatment of choice. In unresectable cases hepatic artery embolization will decrease the risk of variceal hemorrhage.

KEY WORDS: Liver neoplasms, arteriovenous fistula, hepatic resection, embolization, therapeutic

### INTRODUCTION

Shunts between the hepatic artery and the portal vein are present in most malignant liver neoplasms[1,2]. They are frequently small and therefore not always seen on arteriograms[3,4]. Large intrahepatic APF were documented angiographically in 10 to 63 per cent of hepatocellular carcinoma (HCC)[5–7]. APF are uncommon in hepatic metastases or benign tumors[3,8,9].

There are few reports dealing with the clinical picture and treatment of APF in liver neoplasms[10,11]. This report presents our results about prognosis of APF in hepatic tumors in relation to treatment.

### MATERIALS AND METHODS

Hepatic angiography was performed in 130 patients with liver tumors. APF were documented in 15 (42%) of 36 patients with HCC and in one (5%) of 22 patients with benign liver neoplasms. There was no APF among 72 patients with hepatic metastases.

Sixteen patients with APF were distributed in three groups in relation to treatment. Six patients with hepato-cellular carcinoma including four with underly-

Address correspondence to: Pavel G. Tarazov, Cent. Res. Inst. Roentgenol. Radiol., ul. Leningradskaja 70/4, Pesochny-2, Leningrad, 189646, USSR

ing cirrhosis received only conservative therapy (Group A). They were all inoperable. Hepatic artery embolization (HAE) was not performed in three cases because of both the massive tumor and poor patients' condition. The three remaining patients were treated before the clinical use of HAE in our hospital. Resection of both the tumor and the APF was performed in four cases of HCC including two with concomitant cirrhosis (Group B). Proximal HAE with Spongostan (Ferrosan, Copenhagen, Denmark) 2 × 2 × 2 mm cubes and home-made steel coils[12] was carried out in six patients of Group C (five with HCC including three with cirrhosis, and one hepatic hemangiomatosis).

### RESULTS

### *Group A*

Clinical evidence of malignant liver neoplasm including abdominal pain, hepatomegaly, and palpable mass was present in all patients. Symptoms of severe hyperkinetic portal hypertension were present in all 6 cases as well. They included cutaneous vascular spiders, extensive gastroesophageal varices, and ascitis. Four patients previously suffered from gastroesophageal or nasal hemorrhage. Three patients were classified as Okuda's stage 2 and the remaining three as Okuda's stage 3 liver tumor[13]. Angiography showed advanced neoplasms occupying 30 to 80 per cent of the liver, and massive APF with early opacification of the main portal trunk (Figure 1).

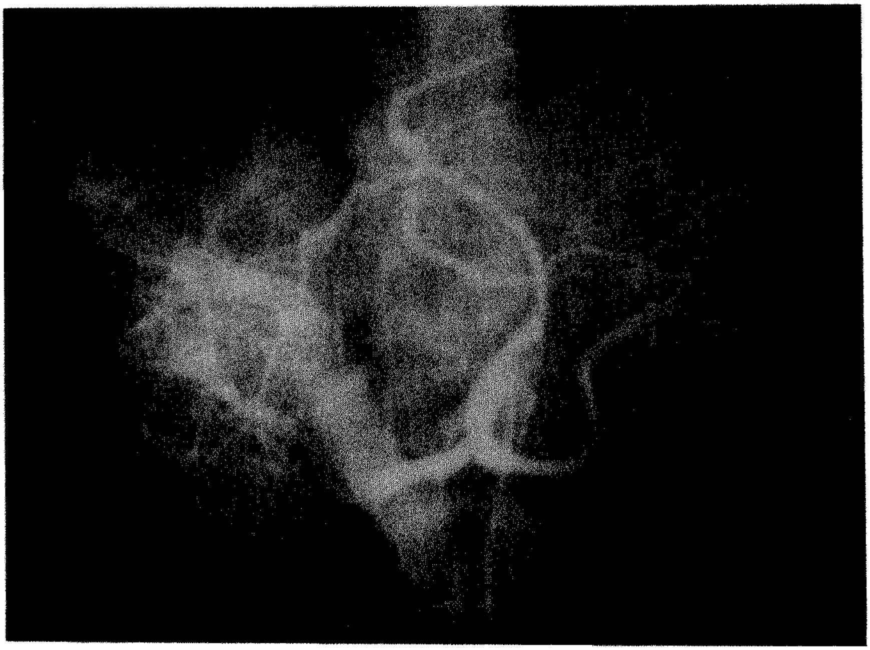

**Figure 1** Celiac angiography of the patient with hepatoma on cirrhosis shows malignant tumor occupying 80% of the liver, and large APF. Portal vein flow is slowed-up. The patient died 3 days after angiography from variceal bleeding.

In spite of conservative therapy, portal hypertension and hepatic insufficiency progressed. All patients died of variceal gastroesophageal bleeding in 3–60 (30.8 ± 21.8) days after angiography.

*Group B*

Hepatic resection was performed in 4 patients with tumors occupying 10 to 30 per cent of the liver. Three of them had Okuda's stage 1 and the remaining one Okuda's stage 2 hepatic tumor. Endoscopy showed gastroesophageal varices of moderate size in all cases. Ascites was present in three patients. Two patients had episodes of nasal hemorrhage. Angiography showed absence of significant portal vein thrombosis and presence of intrahepatic APF of various size. The right main portal branch in three cases and the main portal trunk in the remaining case were seen (Figure 2).

Right hepatectomy in two cases and trisegmentectomy in two patients was carried out. There was no mortality or complications after surgery. All patients noted improvement of overall condition and disappearance of their complaints. Control endoscopy showed complete absence of varices. Ascites disappeared in all three cases as well.

Now two patients are alive and asymptomatic 13 and 29 months after hepatic resection. Recurrence of the tumor but without arteriportal shunting appeared in one patient 44 months later. Peripheral HAE was twice performed, and the patient is well with 50 per cent tumor decreases 52 months after surgery. The remaining patient died 10 months later from lung metastases.

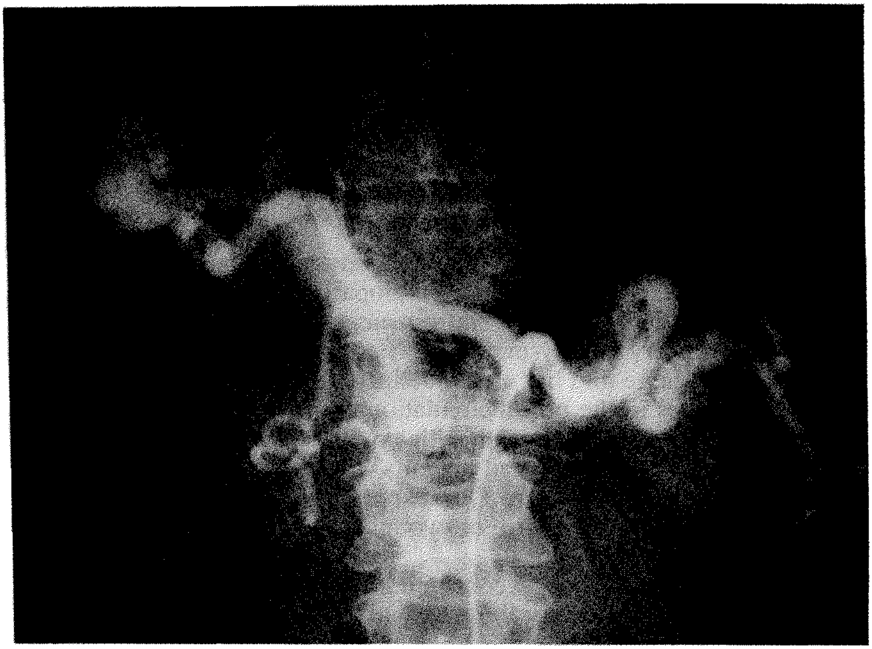

**Figure 2** Celiac angiography of the patient with hepatocellular carcinoma reveals the tumor and large APF in upper liver segments. The patient is well 29 months after hepatic resection.

*Group C*

Proximal embolization of the hepatic artery was performed in 6 patients. Clinical and radiological investigation showed unresectable tumors (five HCC and one capillary hemangiomatosis) with extensive gastroesophageal varices in all cases and ascites in five cases. Four patients experienced gastroesophageal or nasal hemorrhage. Angiography confirmed the existence of a malignant or benign tumor occupying 30 to 60 per cent of the liver, and massive APF with opacification of the main portal trunk in three cases and the right or left portal vein in three cases. Four patients were classified as Okuda's stage 2 and two as Okuda's stage 3 hepatic tumor.

The Seldinger technique via the femoral or left axillary artery was used for catheterization of the hepatic artery. Occlusion of the right (3), right and proper (2), proper and common (1) hepatic arteries with Spongostan cubes and coils was performed (Figure 3a,b). We did not use Ivalon or other small particles because of both the presence of arterioportal shunting and the risk of hepatic failure through massive ischemic necrosis of the tumor.

A postembolization syndrome lasted 7 to 10 days and included abdominal pain, nausea, vomiting, fever, and increased level of liver enzymes. There was partial tumor decrease in 5 patients and no changes in one remaining patient 15–30 days later.

In this group, three patients died in 5, 6 and 30 months respectively after HAE from progression of the neoplasm without evidence of bleeding. One patient with hepatic hemangiomatosis was asymptomatic up to her death from pelvic bone sarcoma three years later. The two remaining patients died from variceal gastroesophageal hemorrhage 2 and 4 months after HAE. These deaths could be explained by rearterialization of the liver via collaterals and restoration of APF.

In total, bleeding from varices developed in 2 of 6 patients after HAE.

### DISCUSSION

Intrahepatic APF are present in most primary malignant liver tumors[1–6]. Alternatively, APF may be congenital[14,15], idiopathic[16], or occur as a consequence of blunt or penetrating, including iatrogenic, trauma[17–20], hepatic cirrhosis[8,9], rupture of hepatic artery aneurysm[3,21,22].

When the APF is large and has a well-defined image on angiograms, significant hemodynamic changes in the portal circulation develop, such as diversion of flow and appearance of portal hypertension with extensive gastroesophageal varices[11,23]. Variceal bleeding occurs in almost all patients with both HCC and APF; conservative treatment is not effective[10,11]. Our experience confirmed these data: fatal hemorrhage developed in the course of two months in all 6 patients receiving only symptomatic therapy.

In our series, 9 of 16 patients had concomitant hepatic cirrhosis. It was difficult to determine whether the cirrhosis or the APF had been more responsible for elevated portal pressure. However, presence of portal hypertension in all patients without cirrhosis showed that APF had a leading role in the development of extensive esophageal varices.

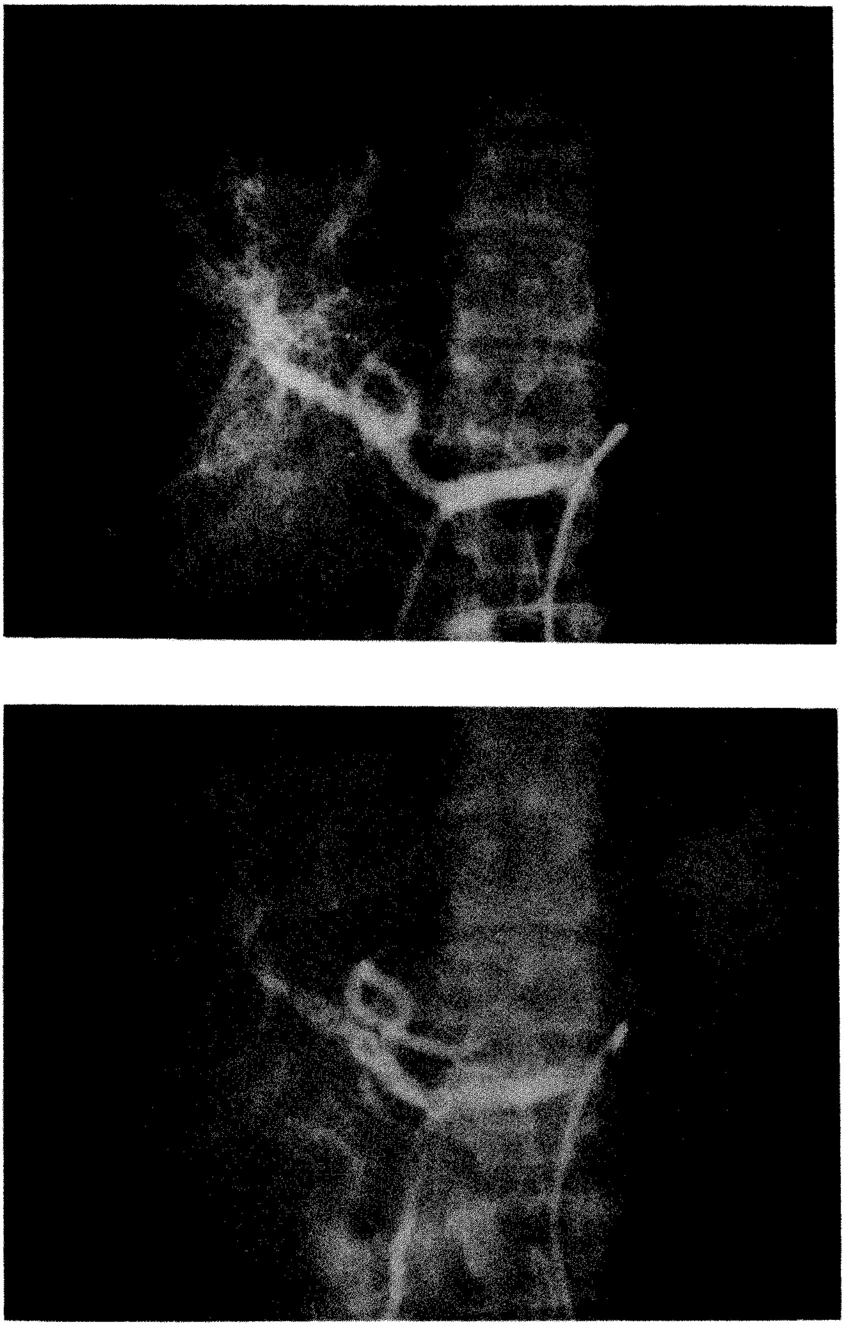

**Figure 3** (a) Hepatic angiography of the patient with hepatoma on cirrhosis shows malignant tumor and APF in the right liver lobe. (b) After embolization of the right hepatic artery APF does not fill up. The patient died 30 months later from tumor progression.

It was interesting that the extent of APF correlated with tumor volume. All patients of Group A had arterioportal shunting with early opacification of the main portal trunk. On the other hand, only the right or left main portal branch was seen on angiograms in 3 of 4 Group B patients and in 3 of 6 Group C patients. The site of conjunction between an enlarged nutritive artery and the portal vein as well as the true size of APF itself could be accurately determined in only two patients of Group B (Figure 2). In the remaining 14 cases multiple small anastomoses within the neoplasm were seen.

Thus, it is possible to state that APF in both malignant and nonmalignant liver diseases need special management. Hepatic resection is the treatment of choice[10,21,24], this is born out by our results: three of four patients are still alive 13–52 months after surgery.

However, radical resection is often impossible because of an advanced tumor. Interruption of hepatic arterial flow to the fistula is necessary in such cases. Nagasue *et al.*[10] recommended hepatic artery ligation for the triad including HCC, gastroesophageal varices, and APF. Mays[25] had the same view and affirmed that APF (excluding iatrogenic) did not disappear spontaneously.

Kim *et al.*[26] considered that a large APF was always life-threatening from rupture or progression of portal hypertension with variceal bleeding. The authors described HAE in three patients with large non-tumor APF and recommended that method as an alternative to surgical ligation. Other investigators have presented similar results[18,19,23,27].

Morse *et al.*[11] performed HAE in four patients with both HCC and APF, and thus normalized hepatic hemodynamics and re-established hepatopetal portal blood flow. Variceal hemorrhage was stopped in all three bleeders. Three patients died 2 to 150 days after HAE, the remaining patient was alive 10 months after embolization without further bleeding. Our results are comparable with this data. One patient died 30 months after HAE and four patients died in the course of 6 months, but only two of variceal hemorrhage. One remaining patient died of a tumor unrelated with hepatic hemangiomatosis and APF.

Various embolic materials were used for embolization of APF: steel coils, Gelfoam and Ivalon particles, isobutyl-2-cyanoacrylate[11,17,23,27]. The authors usually performed superselective embolization of APF. When large APF is revealed on angiograms, we consider that only proximal HAE should be used. Usage of small particles or powders increases the risk of postembolic complications because of their possible passage into the portal circulation. Besides, proximal HAE despite its lesser effectiveness (if compared with peripheral HAE) allows treatment of the tumor and APF simultaneously and to obtain remission in some cases.

Significant systemic hemodynamic changes including reduction of blood pressure, increase in pulse rate and hyperdynamic cardiac failure in infantile hepatic hemangioendothelioma have been reported[28–30]. However, no serious cardiovascular changes were detected in patients with malignant and nonmalignant intrahepatic APF[10,11,19,23]. Also we did not see any evidence of an APF influence on central hemodynamics, even in cases of massive arterioportal shunting. Normalization of both the slightly reduced arterial blood pressure and the moderate tachycardia in two patients of Group B and three patients of Group C could be explained by general improvement with decreased intoxication due to the good therapeutic effect of the treatment.

The investigation of portal pressure before and after surgery and HAE would be interesting, but we considered that such direct invasive measurements were contraindicated in our patients, most of whom were in poor condition.

Information in the literature about effectiveness of HAE in malignant APF is limited. Our groups of patients are small and have considerable differencies in tumor volume and stage of liver involvement. That is why the results of treatment is not fully comparable. Further investigations are needed. However, it is possible to state that prognosis for patients with hepatic tumors complicated by APF is poor because of development of hyperkinetic portal hypertension and subsequent variceal gastroesophageal bleeding. The treatment depends on the extent of liver involvement. Resection of the neoplasm together with the APF is the treatment of choice. In unresectable cases hepatic artery embolization decreases the risk of variceal hemorrhage.

*Acknowledgement*

We thank Greg V. Stiegman, MD (University of Colorado, USA), and George D. Rochlin, MD (Cent. Res. Inst. Roentgenol. Radiol., Leningrad, USSR), for their critical reading of the manuscript and helpful suggestions. We also thank Oleg V. Prijemchenco for preparation of the photographs.

*References*

1. Bookstein, J.J., Cho, K.J., Devis, G.B. and Dail, D. (1982) Arterioportal communications: observations and hypotheses concerning transsinusoidal and transvasal types. *Radiology,* **142**, 581–590
2. Lin,G., Lunderquist, A., Hägerstrandt, L. and Boijsen, E. (1984) Postmortem examination of the blood supply and vascular pattern of small liver metastases in man. *Surgery,* **96**, 517–526
3. Adler, J., Goodgold,M., Mitty, H., Gordon, D. and Kinkhabwala, M. (1978) Arteriovenous shunts involving the liver. *Radiology,* **129**, 315–322
4. Itai, Y., Furui, S., Ohtomo, K., Kokubo,T., Yamauchi, T., Minami, M. and Yashiro, N. (1986) Dynamic CT features of arterioportal shunts in hepatocellular carcinoma. *American Journal of Roentgenology,* **146**, 723–727
5. Okuda, K., Musha, H., Yamasaki, T., Jinnouchi, S., Nagasaki, Y., Kubo, Y., Shimokava, Y., Nakayama, T., Kojiro, M., Sakamoto, K. and Nakashima, T. (1977) Angiographic demonstration of intrahepatic arterio-portal anastomoses in hepatocellular carcinoma. *Radiology,* **122**, 53–58
6. Nakayama, T., Hiyama, Y., Ohnishi, K., Tsuchiya, S., Kohno, K., Nakajima, Y. and Okuda, K. (1983) Arterioportal shunts on dydnamic computed tomography. *American Journal of Roentgenology,* **140**, 953–957
7. Mathieu, D., Grenier, P., Larde, D. and Vasile, N. (1984) Portal vein involvement in hepatocellular carcinoma: dynamic CT features. *Radiology,* **152**, 127–132
8. Itzchak, Y., Adar, R., Bogokowski, H., Mozes, M. and Deutsch, V. (1974) Intrahepatic arterial portal communications: angiographic study. *American Journal of Roentgenology,* **121**, 384–387
9. Heaston, D.K., Chuang, V.P., Wallace, S. and de Santos, L.A. (1981) Metastatic hepatic neoplasms: angiographic features of portal vein involvement. *American Journal of Roentgenology,* **136**, 897–900
10. Nagasue, N., Inokuchi, K., Kobayashi, M. and Saku, M. (1977) Hepatoportal arteriovenous fistula in primary carcinoma of the liver. *Surgery, Gynecology & Obstetrics,* **145**, 504–508
11. Morse, S.S., Sniderman, K.W., Galloway, S., Rapoport, S., Ross, G.R. and Glickman, M.G. (1985) Hepatoma, arterio-portal shunting and hyperkinetic portal hypertension: therapeutic embolization. *Radiology,* **155**, 77–82
12. Ryzhkov, V.K., Borisova, N.A., Gapchenco, E.M. and Dmitrijeva, I.A. (1988) Verschluss von Arterien durch vergrösserte Spiralembolisate. *Radiologia Diagnostica (Berl),* **29**, 713–717

13. Okuda, K., Obata, H., Nakajima, Y., Ohtsuki,T., Okazaki, N. and Ohnishi, K. (1984) Prognosis of primary hepatocellular carcinoma. *Hepatology,* **4**, Suppl 3S–6S
14. Martin, L.W., Benzing, G. and Kaplan, S. (1965) Congenital intrahepatic arteriovenous fistula: report of a successfully treated case. *Annals of Surgery,* **161**, 209–212
15. Helikson, M.A., Shapiro, D.L. and Seashore, J.H. (1977) Hepatoportal arteriovenous fistula and portal hypertension in an infant. *Pediatrics,* **60**, 921–924
16. Mallarini, G., Saitta, S,. Cariati, M., Nicorelli, M. and DeCaro, G. (1982) Embolization of AV intrahepatic fistulas. *European Journal of Radiology,* **2**, 152–156
17. Gmeinweiser, J., von Einsiedel, H., Mix, C. and Dörrler, J. (1989) Ballonokklusion einer intrahepatischen arterio-portalen Fistel. *Fortschritte Röntgenstrahlen,* **150**, 216–218
18. Schmidt, B., Bhatt, G.M. and Abo, M.N. (1980) Management of posttraumatic vascular malformations of the liver by catheter embolization. *American Journal of Surgery,* **140**, 332–335
19. Borisova, N.A., Ryzhkov, V.K., Borisov, A.E. and Gapchenco, E.M. (1989) Arterioportale Fisteln als Folge einer perkutanen transhepatischen Katheterisierung der Vena portae. *Radiologia Diagnostica (Berl),* **30**, 165–170
20. Isik, F.F., Greenfield, A.J., Guben, J., Birkett, D. and Menzoian, J.O. (1989) Iatrogenic arterioportal fistulae: diagnosis and management. *Annals of Vascular Surgery,* **3**, 52–55
21. Van Way, C.W., Grane, J.M., Riddell, D.H. and Foster, J.H. (1971) Arteriovenous fistula in the portal circulation. *Surgery,* **70**, 876–890
22. Langkau, G., Ellinghaus, L. and Müller-Wiefel, H. (1990) Oesophagus-Varicenblutung infolge hepatico-portaler arterio-venöser Fistel durch Perforation eines Aneurysmas der Arteria hepatica. *Chirurg,* **61**, 192–195
23. Rösch, J., Putnam, J.S. and Keller, F.S. (1988) Diagnosis and management of hemobilia. *Seminars in Interventional Radiology,* **5**, 49–60
24. Strodel, W.E., Eckhauser, F.E., Lemmer, J.H., Whitehouse, W.M. and Williams, D.M. (1987) Presentation and perioperative management of arterioportal fistulas. *Archives of Surgery,* **122**, 563–571
25. Mays, E.T. (1977) Vascular occlusion. *Surgical Clinics of North America,* **57**, 291–323
26. Kim, D., Guthaner, D.F., Walter, J.F. and Ryle, R. (1984) Embolization of visceral arteriovenous fistulas with a modified steel wire technique. *American Journal of Roentgenology,* **142**, 1215–1218
27. Clark, R.A., Frey, R.T., Colley, D.P. and Eiseman, W.R. (1981) Transcatheter embolization of hepatic arteriovenous fistulas for control of hemobilia. *Gastrointestinal Radiology,* **6**, 353–356
28. DeLorimer, A.A., Simpson, E.B., Baum, R.S. and Carlsson, E. (1967) Hepatic artery ligation for hepatic hemangiomatosis. *New England Journal of Medicine,* **277**, 333–337
29. Johnson, D.H.., Vinson, A.M., Wirth, F.H., Presberg, H.J., Harkins, G., Nuss, D., Walburgh, C.E. and Raff, J.C. (1984) Management of hepatic hemangiendotheliomas of infancy by transarterial embolization: a report of two cases. *Pediatrics,* **73**, 546–549
30. Becker, J.M. and Heitler, M.S. (1989) Hepatic hemangioendotheliomas in infancy. *Surgery, Gynecology & Obstetrics,* **168**, 189–200

*(Accepted by S.Bengmark 3 April 1991.)*

